# Behavioral Effects and Analgesic Profile of Hemoglobin-Derived Valorphin and Its Synthetic Analog in Rodents

**DOI:** 10.3390/biomedicines11102783

**Published:** 2023-10-13

**Authors:** Petar Todorov, Borislav Assenov, Dimo Angelov, Elena Dzhambazova, Daniela Pechlivanova

**Affiliations:** 1 Department of Organic Chemistry, University of Chemical Technology and Metallurgy, 1756 Sofia, Bulgaria; pepi_37@abv.bg; 2 Institute of Neurobiology, Bulgarian Academy of Sciences, Acad. G. Bonchev Str., Bl. 23, 1113 Sofia, Bulgaria; b.assenov@abv.bg (B.A.); dimo_angeloff@yahoo.com (D.A.); 3 Faculty of Medicine, Sofia University “St. Kliment Ohridski”, 1 Kozyak Str., 1407 Sofia, Bulgaria; elena.dzambazova@abv.bg

**Keywords:** hemorphins, phosphopeptide analog of valorphin, antinociception, inflammation, behavior, opioids

## Abstract

Valorphin (V1) is a naturally occurring peptide derived from hemoglobin that has been found to have an affinity for opioid receptors and exhibits antinociceptive and anticonvulsant activity. Some of its synthetic analogs containing an aminophosphonate moiety show structure-dependent potent antinociceptive effects. This study aimed to reveal a detailed picture of the antinociceptive mechanisms and behavioral effects of V1 and its recently synthesized phosphopeptide analog V2p in rodents using a range of methods. The studied peptides significantly reduced acute (mean V1–9.0, V2p–5.8 vs. controls–54.1 s) and inflammatory (mean V1–57.9 and V2p–53.3 vs. controls–107.6 s) nociceptive pain in the formalin test, as well as carrageenan-induced hyperalgesia (mean V1–184.7 and V2p–107.3 vs. controls–61.8 g) in the paw pressure test. These effects are mediated by activation of opioid receptors with a predominance of kappa in V1 antinociception and by delta, kappa, and mu receptors in V2p-induced antinociception. Both peptides did not change the levels of TNF-alpha and IL-1-beta in blood serum. V1 induces depression-like behavior, and V2p shows a tendency toward anxiolysis and short-term impairment of motor coordination without affecting exploratory behavior. The results characterize valorphin and its derivative as promising analgesics that exert their effects both centrally and peripherally, without causing severe behavioral changes in experimental animals. These encouraging data are a foundation for future studies focusing on the effects of hemorphins after long-term treatment.

## 1. Introduction

The use of opioid substances to treat moderate-to-severe pain has a centuries-old history, and the mechanisms mediating their therapeutic and adverse effects on the body are gradually being revealed at the systemic, cellular, and molecular levels. There are four typical opioid receptors, designated as mu-, delta-, kappa-, and NOPR, which exhibit different selectivity for endogenous and exogenous ligands, different neuroanatomical distributions and network connectivity, and different physiological and behavioral profiles [1,2]. Opioid receptors are distributed throughout the central nervous system (CNS), with high density in the periaqueductal gray (PAG), locus ceruleus, rostral ventral medulla, and substantia gelatinosa of the dorsal horn of the spinal cord, and varying densities in peripheral tissue. Their endogenous ligands are endorphins, enkephalins, dynorphins, endomorphins, and nociceptin. All of these are peptides and, except for nociceptin, contain an “opioid motif” (Tyr-Gly-Gly-Phe-Met/Leu) at the amino terminus, which shows relative selectivity for a particular type of opioid receptor [3]. Along with “classical” opioid receptor ligands, many molecules have been found to possess binding sites and can act as opioid receptor agonists or antagonists. Recently, a new generation of opioid analgesics has been developed that show a high affinity for several opioid receptors, combining the mechanisms that provide high analgesic potential with low levels of tolerance and dependence [4].

Hemorphins are a family of hemoglobin-derived peptides containing 4 to 10 amino acid residues that, according to Nyberg et al., share a common Tyr-Pro-Trp-Thr tetrapeptide core and possess an affinity to opioid receptors [5]. They exert a variety of biological activities under different physiological or pathophysiological conditions. Some of their effects have prompted scientists to expand the study of hemorphins with different peptide chain lengths, as well as their synthetic analogs, to find molecules with therapeutic potential that are more effective and more resistant to enzymatic degradation [6,7].

The first evidence of the opioid-like action of hemorphin-4 and hemorphin-5 isolated from bovine blood provided data for their naloxone-sensitive effects on the in vitro preparation of guinea pig ileum [8]. These peptides were found to be relatively stable against enzymatic degradation in blood plasma, making them interesting targets for further research. Studies have shown that the short-chain hemorphins bind with high affinity to mu- and delta-opioid receptors and with less affinity to kappa receptors [9]. Furthermore, endogenous opioid peptides, including hemorphin, were found to act as both agonists and antagonists depending on the tissue-specific or tolerance-dependent differences in the number and type of opioid receptors [10,11]. Based on their N-terminal sequences, the latter peptides are classified as hemorphins, V-hemorphins, VV-hemorphins, and LVV-hemorphins [12,13]. Similar to the tetra- and pentapeptides, longer N-terminally extended hemorphin peptides, such as VV-hemorphin-7 and LVV-hemorphin-7, also exhibit opioid activity in the rat brain [14]. An earlier study has suggested that the antinociceptive effect of hemorphin-4 and -5 is related to a free terminal amino acid tyrosine that was considered important in opioid receptor interactions [15]. The heptapeptide valorphin (Val-Val-Tyr-Pro-Trp-Thr-Gln) is a hemorphin produced by the proteolytic cleavage of the region 33–39 of the β-globin chain of hemoglobin, which has an N-terminal extension but demonstrated a reduced C-terminal and a lower affinity for delta- than for mu-opioid receptors as compared with hemorphin-4 [16]. Our research team has pioneered studies on the synthesis of hemorphin analogs obtained by substitution with unnatural amino acids and/or steric-restricted amino acids or conjugation with other molecules in their structure. We have shown that these can activate the brain inhibitory pain system, producing dose-dependent antinociception in experimental models of phasic and tonic pain in rodents, as well as significant anticonvulsant activity [17,18,19]. Among the most promising in terms of their antinociceptive profile were hemorphin peptide analogs containing amino phosphonate moiety [18].

This study aimed to reveal a detailed picture of the antinociceptive mechanisms and behavioral effects of valorphin (V1) and its recently synthesized phosphopeptide analog V2p.

## 2. Materials and Methods

### 2.1. Synthesis

**Figure 1:**
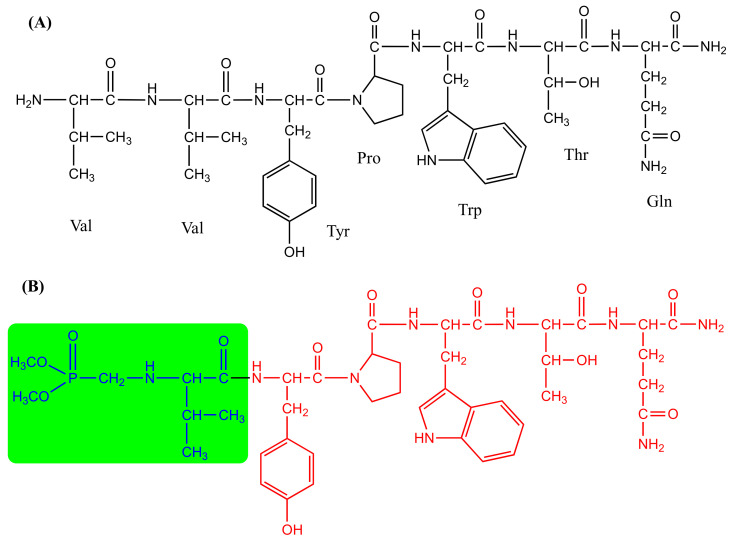
Chemical structures of investigated hemorphin opioid peptides. (**A**) Amideted valorphin V1 and (**B**) newly synthesized V2p: valorphin containing aminophosphonate moiety (in green) at N-terminal.

Each one of the chemicals and solvents was of analytical or HPLC quality, bought from Fluka or Merck, and was utilized unpurified. Valorphin analog containing aminophosphonate moiety at N-terminal–V2p (Figure 1), was prepared as per our recently described procedure [19]. All the physicochemical and analytical data of the compound were identical to those previously described [19].

### 2.2. Biological Assay

#### 2.2.1. Experimental Animals and Drug Treatment

Adult male ICR mice (weighed 25–30 g) and Wistar rats (weighed 250–300 g) obtained from the Animal Facility of the Institute of Neurobiology, were accommodated for a week in transparent standard cages (5 per cage) at room temperature of 21 ± 2 °C; under 12/12 h light/dark cycle and with access to pellets and water ad libitum except during the test. Animals were randomly divided into groups of *n* = 8. The valorphin and its analog were dissolved in sterile saline and injected intracerebroventricularly (ICV) at doses of 50, 25, and 12.5 µg/5 µL/mouse, according to the procedure described elsewhere or intraplantar at a dose of 50 µg/5 µL/paw in rats [19,20,21]. ICV injections are performed using a 28-gauge stainless steel needle attached to a 10 μL Hamilton^®^ syringe (Lidingö, Sweden) fitted with a 3 mm stop on the needle, and the puncture site was according to the coordinates of the right lateral ventricle [22]. The opioid receptor antagonists naltrindole (1 mg/kg), nor-binaltorphimine (1 mg/kg), and naloxone (5 mg/kg) were injected intraperitoneally (IP) 15 min before the test peptide and 30 min for nor-binaltorphimine. The reference drugs morphine and indomethacin (5 mg/kg) were injected IP 15 min before the start of the test. Drugs were purchased from Merck, Darmstadt, Germany, dissolved in sterile saline, and injected in a volume of 10 mL/kg body weight. Serum levels of tumor necrosis factor-alpha (TNF-α) and interleukin-1 beta (IL-1β) were measured using commercially available enzyme-linked immunosorbent assay (ELISA) kits (IBL International, Hamburg, Germany) according to the manufacturer’s protocol. Their concentration was quantified using an ELISA reader (ELx800 Absorbance Reader, BioTek Instruments Co., Winooski, VT, USA).

All animal experiments were conducted in accordance with the Declaration of Helsinki Guiding Principles on Care and Use of Animals (DHEW Publication, NHI 80-23) and EC Directive 2010/63/EU for animal experiments. The protocols implemented in this study were approved by the Ethics committee of the Bulgarian Food Safety Agency (No. 176/2019).

#### 2.2.2. Experimental Design

Experiment 1: Test peptides were injected in three doses before the formalin test to investigate the effective dose on both acute and inflammatory tonic pain. Immediately after the end of the test, the mice were decapitated, and the blood was collected in tubes with a coagulation accelerator to obtain blood serum. Serum samples were assayed by ELISA for tumor necrosis factor-alpha levels according to the manufacturer’s protocol. In separate groups, the selected dose of 25 µg/mouse was injected 15 min before the “Rota-rod” test, the “Elevated plus maze” test, and the “Tail suspension” test to study their effects on motor coordination, anxiety-like behavior, and depressive-like behavior, respectively. Additional groups of mice were coadministered the selective delta-, kappa- and mu-opioid receptor antagonists naltrindole, nor-binaltorphimine (nor-BNI), and naloxone before the investigated peptides (25 µg/mouse) to assess their opioidergic mechanism of action.

Experiment 2: The local effects of valorphin and its analog were assessed in rats. Test peptides were injected locally intraplantar before the primary measurement of mechanical pain threshold as well as paw volume, both of which were measured before and at 1, 3, and 4 h after the induction of local inflammation by intraplantar carrageenan injection. After the 4th hour, the rats were sacrificed, and the blood was collected in tubes to separate the serum. Serum samples were analyzed by ELISA for interleukin-1 (IL-1) beta levels according to the manufacturer’s protocol.

#### 2.2.3. Methods

1. Formalin test

Mice were placed into a transparent cage (15.0 cm × 15.0 cm × 12.5 cm high), which also served as an observation chamber, and were allowed to adapt to their environment for 1 h before testing. Five min after ICV injections the plantar surface of the hind paw was injected subcutaneously (SC) with 10 μL of 5% formalin. Time spent licking the injected paw was measured 40 min after formalin injection in two intervals corresponding to the two phases of the test: early 0–20 min and late inflammatory from 20–40 min. A mirror was positioned at a 45° angle below the floor allowing an unobstructed view of the animal’s hind paw.

2. Rota-rod test

Motor coordination was assessed in mice by the rotary rod test. The apparatus consisted of a rotating rod (3.2 cm in diameter, at a speed of 8 rpm) raised 30 cm above the ground, where each mouse was tested to remain on the moving rod without falling for up to five minutes at the 10, 25 and 40 min after peptide injection. Latency to fall was additionally calculated.

3. Elevated plus maze (EPM)

The apparatus used for the elevated plus maze test comprises two open arms (25 × 5 cm) across from each other and perpendicular to two closed arms (25 × 8 × 14 cm) with a center platform (5 × 5 cm). The entire apparatus is 18 cm above the floor and is placed in an empty circular tank (100 cm diameter, 35 cm tall) to protect the mice that fall or attempt to escape during the experiment. The apparatus is made of wood and painted in black.

4. Tail suspension test

The test involves suspending mice above the ground by their tails in suspension boxes made of black plastic (55 height × 60 width × 20 cm depth). The paper tape adheres tightly to the tail of the mouse at 2 cm and to the suspension bar at 15 cm. Transparent hollow polycarbonate cylinders (4 cm length, 1.3 cm inner diameter, 1 g) were placed around the tails of the mice to prevent any tail-climbing behavior. Mice are placed in this inescapable but moderately stressful situation for 6 min. Lack of escape-related behavior is considered immobility [23].

5. Carrageenan-induced hyperalgesia and edema

Paw edema was induced by λ-carrageenan mixed in saline (CRG, Sigma Aldrich; 1% *w*/*v* in saline, 10 mg/mL) injected 0.1 mL intraplantarly into the right hind paw of a rat [24]. Peptides were injected intraplantarly into the same paw, 5 min before CRG application. Mechanical pain threshold, as well as paw volume, were measured before and at 1, 3, and 4 h after CRG injection using an Ugo Basile (Gemonio, Italy) analgesimeter and plethysmometer, respectively. The minimum pressure (in grams) that elicits a pain response, such as withdrawal or struggle, is defined as the pain threshold. Paw volume was measured in mL and expressed as a change from baseline volume before the edema [25].

#### 2.2.4. Data Analysis

Results were calculated and visualized by Sigma Stat v11.0 and Sigma Plot v11.0. Data were analyzed by one-way ANOVA with the factors “drug” or “dose” and two-way ANOVA with repeated measurements with factors “drug” and “time”, followed by Tukey’s post hoc test. Values are mean ± SD, where *p* < 0.05 was considered statistically significant.

## 3. Results

### 3.1. Experiment 1

**Figure 2:**
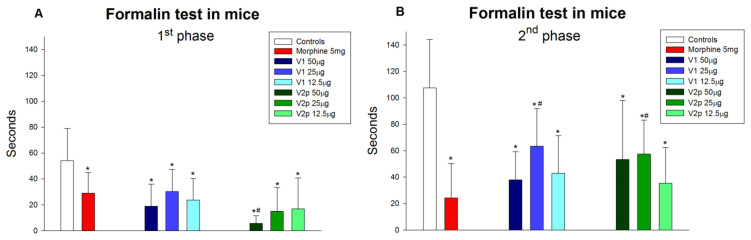
The effects of valorphin (V1), its phosphonate analog (V2p), and the reference drug morphine on nociception (time to licking the injected paw) during the acute (**A**) and inflammatory (**B**) phases of the formalin test in mice. Each group (*n* = 8) was injected with one dose of the peptide intracerebroventricularly (ICV), and morphine was injected intraperitoneally (IP). Data show the mean ± SD. * *p* < 0.05 vs. controls treated ICV with saline and injected SC with formalin; # *p* < 0.05 compared with morphine.

The precursory peptide valorphin (V1, Val-Val-Tyr-Pro-Trp-Thr-Gln-NH2) showed a significant antinociceptive effect in all applied doses during the 1st phase (F 3, 27 = 5.175, *p* = 0.006) and the 2nd phase (F 3, 27 = 9.327, *p* < 0.001) of the performed formalin test for acute and inflammatory pain (Figure 2A,B). The highest dose (50 µg/5 µL) displayed the most pronounced analgesic effect in both phases of the test, comparable to the effect of the referent drug morphine. The peptide V2p showed a significant antinociceptive effect in all three doses used in the formalin test. During the acute phase, V2p also showed a significant antinociceptive effect in all applied doses (F 3, 27 = 5.175, *p* = 0.006) with the most pronounced analgesic effect at the dose of 50 µg/5 µL (*p* < 0.001). The effect of V2p was significantly stronger compared with the effect of the referent morphine (F 1, 15 = 15.199, *p* = 0.001) (Figure 2A). During the inflammatory phase, the effect of V2p was comparable (F 3, 27 = 4.787, *p* = 0.008), but the most significant effect was shown at the lowest dose–12.5 µg/5 µL (*p* < 0.001) (Figure 2B).

**Figure 3:**
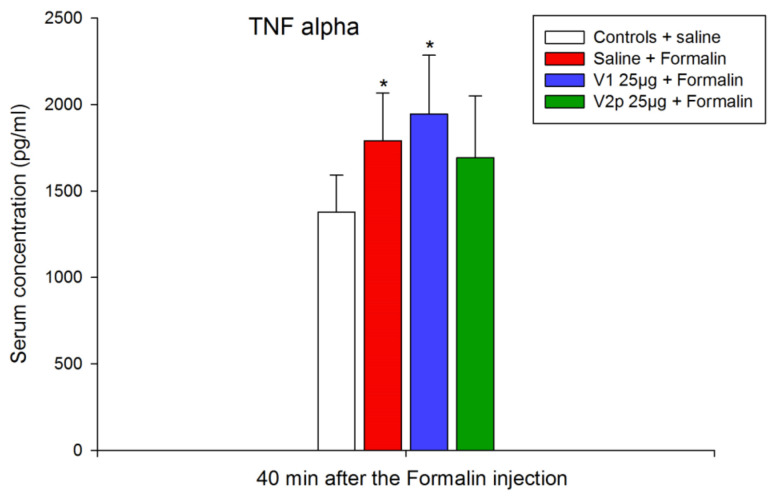
Serum levels of tumor necrosis factor-alpha (TNF-α) in the naive control group (saline), the formalin-injected group (saline + formalin), and the groups injected ICV with valorphin (V1, 25 µg + formalin), or its phosphonate analog (V2p, 25 µg + formalin) and formalin. Data show the mean ± SD; *n* = 8. * *p* < 0.005 vs. controls injected ICV with saline.

Experimental data from the ELISA test showed that formalin treatment increased the blood serum level of TNF-alpha (F 1, 7 = 6.448, *p* = 0.039) and V1 did not change this trend (F 1, 11 = 10.982, *p* = 0.007) but in animals treated with V2p, the concentration of TNF-alpha is comparable to the controls (Figure 3).

**Figure 4:**
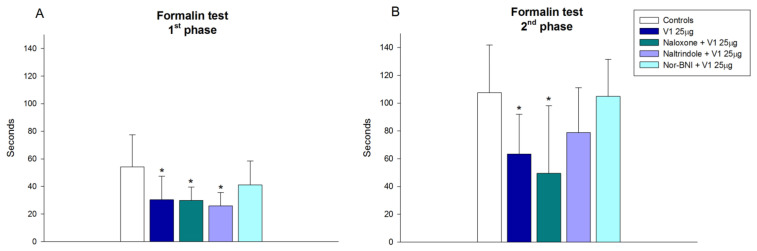
Effects of blocking the opioid receptors µ (naloxone, 5 mg, IP), δ (naltrindole 1 mg, IP), and κ (Nor-BNI, 1 mg, IP), on the antinociception of valorphin (V1, 25 µg, ICV) in acute (**A**) and inflammatory (**B**) phases of formalin test in mice. Data show the mean ± SD; *n* = 8. * *p* < 0.05 vs. controls treated ICV with saline and injected SC with formalin.

We combined three selective opioid receptor antagonists naltrindole (1 mg/kg, IP), nor-BNI (1 mg/kg, IP), and naloxone (5 mg/kg, IP), injected before the average effective dose (25 µg/5 µL) of reference peptide V1 to establish the involvement of opioid receptors in valorphin-induced antinociception. The results show that the kappa opioid receptor antagonist Nor-BNI significantly (*p* < 0.05) abolished V1 antinociception in both phases of the performed test, while other antagonists did not alter the effects of the peptide (Figure 4A,B).

**Figure 5:**
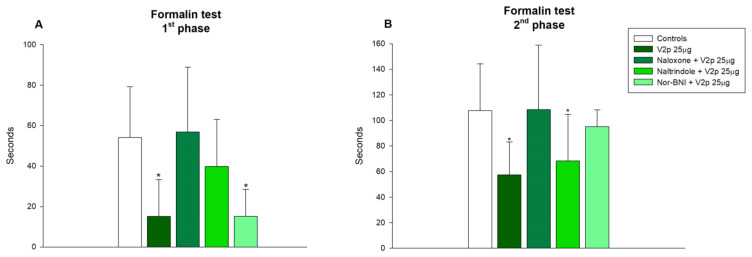
Effects of blocking the opioid receptors µ (naloxone, 5 mg, IP), δ (naltrindole 1 mg, IP), and κ (Nor-BNI, 1 mg, IP), on the antinociception of phosphonate valorphin analog (V2p, 25 µg, ICV) in acute (**A**) and inflammatory phases (**B**) of formalin test in mice. Data show the mean ± SD; *n* = 8. * *p* < 0.05 vs. controls treated ICV with saline and injected SC with formalin.

The combination of the same doses of opioid receptor antagonists with V2p (25 µg/5 µL) showed that naloxone, which has low selectivity in blocking certain opioid receptors, antagonized the antinociceptive effect of V2p in both phases of the formalin test (*p* < 0.05, Figure 5A,B). Delta opioid receptor antagonist naltrindole significantly abolished (F 1, 16 = 5.127, *p* = 0.038) the antinociception of V2p during the acute phase of the test. Nor-BNI significantly diminished (F 1, 15 = 14.442, *p* = 0.002) the antinociceptive effect of V2p on the inflammatory pain (Figure 5B).

**Figure 6:**
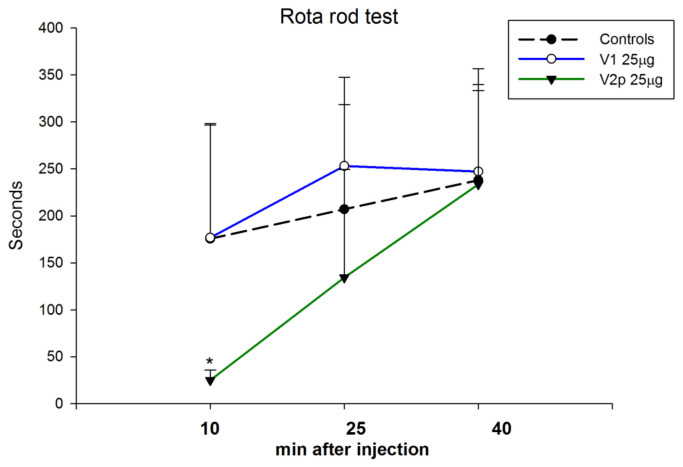
Effects of valorphin (V1, 25 µg, ICV) or its phosphonate analog (V2p, 25 µg, ICV) on motor coordination assessed by the rota-rod test in mice. Data show the mean ± SD. * *p* < 0.05 vs. controls injected ICV with saline.

The results show that V1 did not affect motor coordination in the rotating rod test (Figure 6). However, V2p (25 μg/5 μL) showed a significant impairment of motor coordination at the 10th minute post-injection (*p* < 0.01) and had no effect at subsequent test intervals (factor “time” F 2, 45 = 7.11, *p* = 0.002; factor “drug” F 1, 45 = 6.69, *p* = 0.001) (Figure 6).

**Figure 7:**
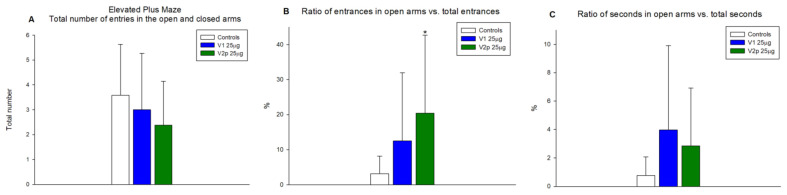
Evaluation of the anxiolytic effect of the tested peptides (V1 and V2p, 25 µg, ICV) in the elevated plus maze in mice. Study activity expressed as the total number of entries in the open and closed arms (**A**). Ratio of entries in open hands as a percentage of total entries. (**B**) Ratio of time spent in open arms as a percentage of total test duration (**C**). Results are expressed as means ± SD; *n* = 8. * *p* < 0.05 vs. controls injected ICV with saline.

**Figure 8:**
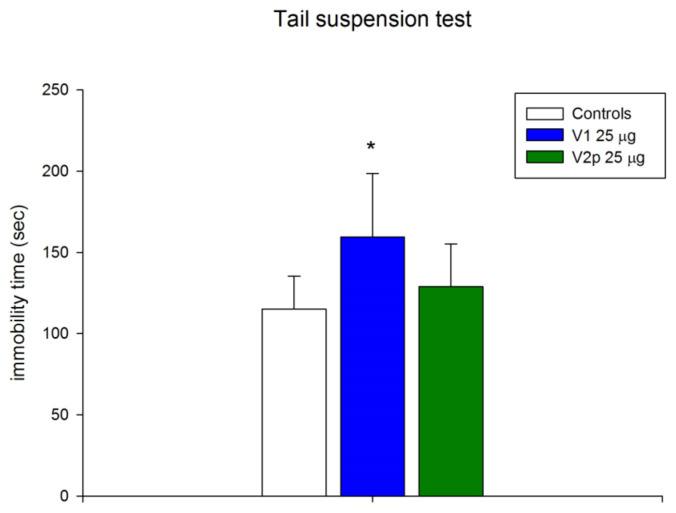
For depression-like behavior (duration of immobility) in the tail suspension test in mice injected with valorphin and its analog (V1 and V2p, 25 µg, ICV). Results are expressed as means ± SD; *n* = 8. * *p* < 0.05 vs. controls injected ICV with saline.

The same doses of the peptides were tested in the “Elevated plus maze” for effects on anxiety-like behavior. The data show that V1 and V2p did not significantly alter the total locomotor and exploratory activity (number of entries in both open and closed arms) compared with controls (Figure 7A). V2p showed a significant anxiolytic effect (F 1, 23 = 8.635, *p* = 0.007) only by an increased number of entries in the open arms of the elevated plus maze, but not in terms of time spent in these open arms (Figure 7B,C). V1 did not induce significant changes in normal anxiety behavior in an unfamiliar environment (Figure 7B,C), however, it produced a depressive-like behavior in the tail suspension test, increasing the immobility time (F 1, 16 = 9.531, *p* = 0.008) as compared with the controls (Figure 8).

### 3.2. Experiment 2

**Figure 9:**
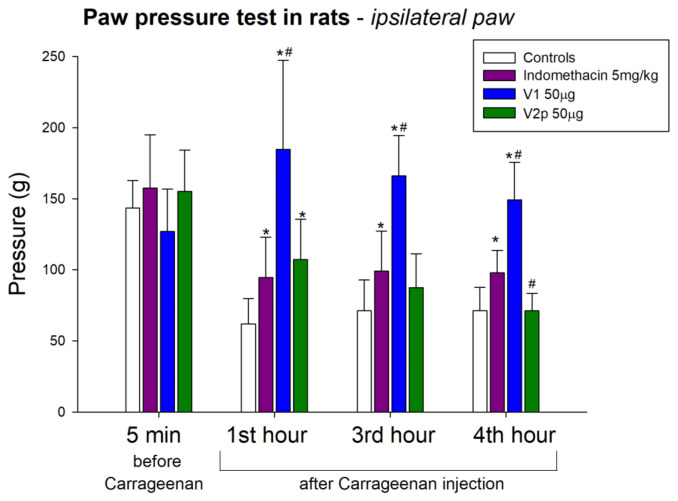
Effects of locally intraplantar-injected valorphin and its analog (V1 and V2p, 50 µg, IPL) on the mechanical nociceptive threshold (pressure in grams) at 5 min after peptide injection and 1, 3, and 4 h after subsequent IPL injection of 1% carrageenan in the same paw. Data show the mean ± SD; *n* = 8. * *p* < 0.05 vs. negative controls with carrageenan; # *p* < 0.05 compared with indomethacin.

The intraplantar injection of V1 at a dose of 50 μg/5 μL had a significant anti-hyperalgesic effect (factor “drug” F 1, 44 = 101.619, *p* < 0.001) on carrageenan-induced inflammatory hyperalgesia with a significance from the 1st to the 4th hours after the irritant injection (factor “time” was insignificant). This effect was relatively constant and significantly stronger than that of the reference drug indomethacin (factor “drug” F 1, 47 = 21.000, *p* < 0.001; “time” was insignificant) (Figure 9). V2p injected at the same dose and route showed a significant effect only in the first hour after carrageenan injection (factor “drug” F 1, 47 = 11.852, *p* = 0.001; interaction “drug” × “time” F 2, 47 = 4.941, *p* = 0.012). At the remaining time points of the experiment (3rd and 4th hour), our results show that intraplantar injection of V2p did not increase the pain threshold (Figure 9).

**Figure 10:**
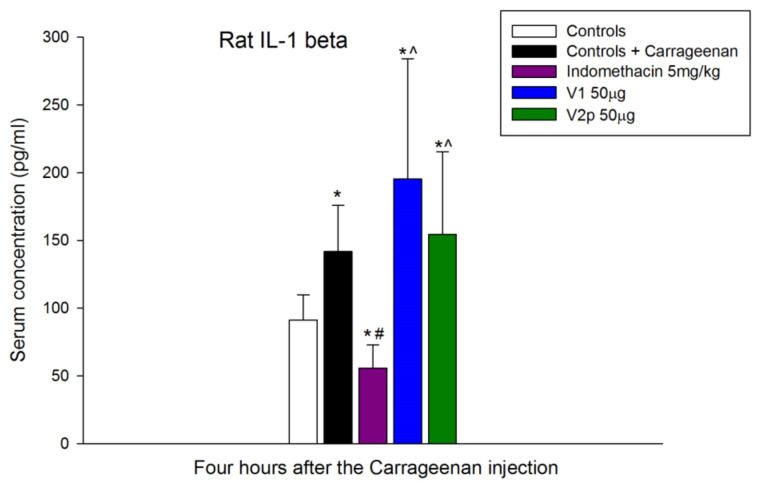
Effects of locally intraplantar-injected valorphin and its analog (V1 and V2p, 50 µg, IPL) on the blood serum level of IL-1, 4 h after a subsequent IPL injection of 1% carrageenan in the same paw. Data show the mean ± SD; *n* = 8. * *p* < 0.05 vs. naïve controls; # *p* < 0.05 vs. carrageenan negative controls; ^ *p* < 0.05 vs. indomethacin positive controls.

ELISA data show that carrageenan 1% significantly increased the serum concentration of IL-1 beta (F 1, 15 = 13.842, *p* = 0.002). Results demonstrate that the injection of peptides V1 (F 1, 12 = 10.718, *p* = 0.007) and V2p (F 1, 12 = 7.839, *p* = 0.016) increased the serum concentration of IL-1 beta compared with the controls as well as the anti-inflammatory drug indomethacin (F 1, 14 = 15.605, *p* = 0.001) (Figure 10).

**Figure 11:**
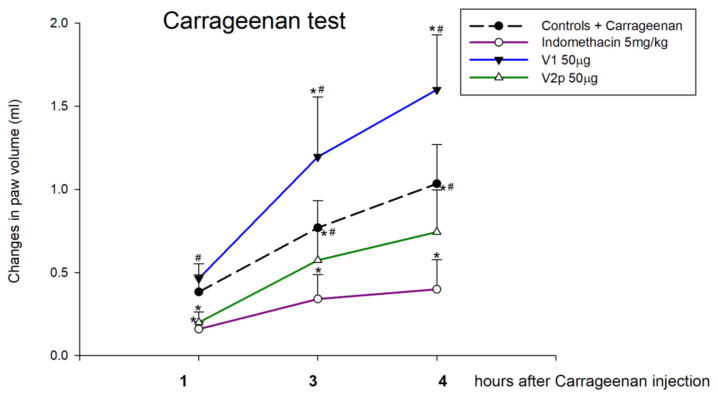
Effects of locally intraplantar-injected valorphin and its analog (V1 and V2p, 50 µg, IPL) on the paw edema (increase in paw volume) 1, 3, and 4 h after a subsequent IPL injection of 1% carrageenan in the same paw. Data show the mean ± SD; *n* = 8. * *p* < 0.05 vs. carrageenan negative controls; # *p* < 0.05 vs. indomethacin positive controls.

Our experimental data show that the local injection of 50 µg/5 µL V1 potentiates the inflammatory changes in the volume of the ipsilateral (carrageenan-injected) paw (factor “drug” F 1, 43 = 20.279, *p* < 0.001; factor “time” F 2, 43 = 43.574, *p* < 0.001; interaction “drug” × “time” F 2, 43 = 3.295, *p* = 0.048) (Figure 11). At the 1st, 3rd, and 4th hour after carrageenan injection the other analyzed peptide, V2p, had a significant anti-inflammatory effect (factor “drug” F 1, 47 = 14.466, *p* < 0.001; factor “time” F 2, 47 = 36.310, *p* < 0.001), but not as effective as the referent drug indomethacin (factor “drug” F 1, 47 = 63.925, *p* < 0.001; factor “time” F 2, 47 = 24.051, *p* < 0.001; interaction “drug” × “time” F 2, 47 = 5.098, *p* = 0.01) (Figure 11).

## 4. Discussion

The main objective of this study was to characterize the antinociceptive and anti-hyperalgesic potential of a newly synthesized valorphin analog with an aminophosphonate moiety at the N-terminus, and to learn more about the mechanisms of valorphin-induced effects on nociceptive and inflammatory pain. As previously observed, both the type of the integrated amino acid and the position of the amino acid replacement in opioid hemorphins significantly alter the valorphin activity and affinity [21]. Previous research on several VV-hemorphin-5 analogs has established their structure–effect link and antinociceptive potential [18,19,20]. C-terminal amide groups in peptide chains have been discovered to be more resistant to enzymatic degradation and to have conformations that are more suited for interaction with specific receptors, allowing them to be suitable for the synthesis of novel peptide analogues [26,27]. Aminophosphonates are gaining increasing research interest as one of the most common organophosphorus derivatives, with various biological activities, including enzyme inhibitors, peptide mimics, antiviral, antibacterial, and anticancer properties, in various applications [28,29]. The N-modified analog of VV-hemorphin-5 bearing an aminophoshonate moiety (V2p) was produced by replacing the two N-terminal Val from the native VV-hemorphin-5 with alpha-aminophosphonate ((dimethoxyphosphoryl)methyl)-L-valine. Figure 1 depicts the chemical structures.

The present data reveal a significant effect of valorphin (V1) as well as its synthetic analog (V2p) in suppressing pain sensitivity at the supraspinal level as well as locally in the inflamed tissue. This study elucidates, for the first time, the involvement of different types of opioid receptors in valorphin-induced antinociception. Although valorphin is a peptide with known antinociceptive effects, the cellular and systemic mechanisms of its activity were not yet fully understood [15,19,20,21,30,31]. Some of the receptor mechanisms of other members of the hemorphin family are reported in the literature. It is thought that the Tyr-Pro-Trp-Thr core in the structure of hemorphins is responsible for their binding to opioid receptors [6]. In support of this assumption, in vitro studies on smooth muscle contraction have shown that hemorphin-4, the shortest member of the hemorphins with this configuration, has inhibitory activity comparable to other opioid peptides acting on the mu-opioid receptor [10]. Further, in silico data have confirmed that the hemorphin interacts with specific amino acid residues of the mu-opioid receptor [30]. This tetrapeptide however has shown nearly equal affinity to mu-, delta- and kappa-opioid receptors in brain cellular membranes, which suggests a multilateral interaction with opioid receptors depending on their local density [9]. Non-opioid mechanisms of hemorphin action also cannot be excluded. Supportive data have shown that a longer hemorphin LVV-H7 attenuated carrageenan-induced hyperalgesia at the spinal cord level in an opioid-independent manner; however, at the supraspinal level, it could produce a naloxone-reversible anti-hyperalgesia [30]. Our present results show that valorphin-induced antinociception during the acute and inflammatory phases of the formalin test are abolished by the kappa-selective antagonist nor-BNI. This evidence demonstrates for the first time the kappa-opioid receptor mechanism of valorphin-induced antinociception, which is consistent with our previous docking analysis data showing that valorphin has a better interaction energy with the kappa opioid receptor and a weaker interaction with the delta opioid receptor [20]. Activation of kappa-opioid receptors, along with other opioid receptors, is known to suppress nociception in the formalin test in mice, and this effect is more pronounced against pain sensitization during the second phase of the test [32,33]. Our data show that the antinociceptive effect of valorphin against inflammatory pain is not correlated with tumor necrosis factor-alpha (TNF-a), hence its blood plasma level was not decreased as compared with the controls treated with formalin.

The present aminophosphonate analog of valorphin (V2p) showed a more complex interaction with opioid receptors. Its strong antinociceptive effect against acute formalin-induced nociception was antagonized by both the delta-receptor antagonist naltrindole and the preferential mu-receptor antagonist naloxone, while, during the second phase, antinociception was completely blocked by nor-BNI and naloxone. The overall effect of V2p in the formalin test in mice is a significant antinociception mediated by all three opioid receptors with a transient effect on delta- and long-term effects due to mu- and kappa-receptors. We can speculate that the structural changes in the valorphin molecule are sufficient to induce a conformational shift allowing increased affinity for the other opioid receptor types. Activation of the brain opioidergic mechanism by valorphin is not accompanied by an anxiolytic effect as shown by normal avoidance of the open arms in the elevated plus maze, although it provokes depression-like behavior expressed by increased immobility in the tail suspension test. In addition, valorphin did not show a sedative effect because it did not impair motor coordination in the rota-rod test. V2p impaired motor coordination only 10 min after its intracerebral injection, followed by recovery. Kappa opioid receptors in the CNS are thought to induce maladaptive sensitivity and dysphoria in stress models, increasing anxiety [34,35,36]. We can speculate that the depressive-like effect of valorphin is related to the activation of brain kappa receptors. However, conflicting data support the idea that kappa receptor activation induces anxiolytic and antidepressant behaviors [36]. The studied peptides showed an antinociceptive effect against carrageenan-induced hyperalgesia after their local injection. The mechanical antinociception is longer lasting for valorphin (more than 4 h) and 1 h for the V2p analog. However, this effect was not associated with any significant changes in elevated serum IL-1-beta levels compared with carrageenan-treated negative controls. Furthermore, our tested peptides showed different effects on the development of edema after carrageenan injection. Thus, valorphin potentiates the progression of edema, while V2p suppresses it. Literature data indicate that hemorphins are involved in the inhibition of hyperalgesia at the spinal and supraspinal levels, but our data demonstrate their local efficacy. LVV-H7 was shown to attenuate carrageenan-induced hyperalgesia at the spinal level and to a lesser extent at the supraspinal level, which could not be reversed by co-administration of naloxone [30]. This anti-hyperalgesic effect of LVV-H7 was found to possess a remarkable duration in contrast with the short-term effects of some other natural peptides. The authors propose that this hemorphin may prevent the development of inflammation-induced central sensitization in the spinal cord [30]. Relatively few data are available on the anti-inflammatory effects of hemorphins when applied topically. Hemorphin-7 significantly reduced both plasma extravasation and vasodilation induced by injection of substance P but did not affect the vasodilation response to CGRP. Naloxone as a general opioid antagonist can reverse the inhibitory effect of hemomorphin-7 on the inflammatory response to substance P [37].

## 5. Conclusions

Our data reveal a significant effect of valorphin as well as its synthetic analog V2p in suppressing both acute pain sensitivity and hyperalgesia. This study elucidated, for the first time, the involvement of specific types of opioid receptors in V2p-induced antinociception. Taken together, the data indicate that valorphin derivatives are a promising group of potential analgesics that exert their effects both centrally and peripherally, without causing severe behavioral changes in experimental animals. These encouraging data are a foundation for future studies focusing on the effects of hemorphins after long-term treatment.

## Data Availability

All data are available by request from the principal author.
